# Screening Older Adult Men for Abdominal Aortic Aneurysm: A Scoping Review

**DOI:** 10.1177/15579883211001204

**Published:** 2021-03-16

**Authors:** Priya Bains, John L. Oliffe, Martha H. Mackay, Mary T. Kelly

**Affiliations:** 1 School of Nursing, University of British Columbia, Vancouver, BC, Canada; 2 Department of Nursing, University of Melbourne, Melbourne, VIC, Australia; 3 Centre for Health Evaluation and Outcomes Sciences, Vancouver, BC, Canada

**Keywords:** Male abdominal aortic aneurysm, screening men for abdominal aortic aneurysm, men’s primary health care

## Abstract

Abdominal aortic aneurysm (AAA) is a potentially fatal condition predominantly affecting older adult men (60 years or over). Based on evidence, preventative health-care guidelines recommend screening older males for AAA using ultrasound. In attempts to reduce AAA mortality among men, screening has been utilized for early detection in some Western countries including the UK and Sweden. The current scoping review includes 19 empirical studies focusing on AAA screening in men. The findings from these studies highlight benefits and potential harms of male AAA screening. The benefits of AAA screening for men include decreased incidence of AAA rupture, decreased AAA mortality, increased effectiveness of elective AAA repair surgery, and cost-effectiveness. The potential harms of AAA screening included lack of AAA mortality reduction, negative impacts on quality of life, and inconsistent screening eligibility criteria being applied by primary care practitioners. The current scoping review findings are discussed to suggest changes to AAA screening guidelines and improve policy and practice.

Aneurysms are defined as an irreversible and focal dilatation of a blood vessel that exceeds 1.5 times the normal vessel diameter (Carino et al., 2018). The aorta is the largest blood vessel in the human body, and it functions to transport oxygenated blood from the heart (Collins et al., 2014). The abdominal aorta has an approximate diameter of 2.0 cm in most adult males, with normal variations due to age, height, and weight (Meyermann & Caputo, 2017).

An abdominal aortic aneurysm (AAA) is defined as an aneurysm of the abdominal aorta ≥3.0 cm or 50% larger than normal abdominal aorta diameter (Kumar et al., 2017). The exact pathogenesis and etiology of AAA formation are considered multi-factorial (Hao et al., 2017; Sakalihasan et al., 2018). The three major pathogenic theories include inflammation, proteolysis (protein degradation), and vascular smooth muscle apoptosis (programmed cell death) (Powell, 2007). Risk factors for AAA development include male sex, older age, tobacco use, family history, European ancestry, hypertension, hypercholesteremia, and history of other large vessel aneurysms (Benson et al., 2018; Carino et al., 2018; Cornuz et al., 2004; Wanhainen et al., 2020). There are conflicting data about atherosclerosis and obesity as risk factors for AAA development (Carino et al., 2018). Screening is key for identification and early intervention to reduce the AAA mortality rate among men. The current scoping review provides a synthesis of the literature to describe benefits and potential harms of AAA screening older men (≥65 years old) residing in North America.

## AAA Epidemiology

Study findings previously reported AAA prevalence between 3.9% and 7.2% in screened men aged 50 and older (Lindholt et al., 2005; Norman et al., 2004); however, current prevalence has been reported at 1.3%–5% of screened men 65 years and older (Wanhainen et al., 2020). In addition, though there has been a reduction in aortic diameter over time in screened men, the growth rates of small and medium AAAs have not declined (Sweeting et al., 2018). Once AAA is detected, the average life expectancy for males is 11 years (Ashton et al., 2007). The most critical risk of living with AAA is the possibility of rupture (rAAA), which carries a mortality rate of up to 81% for men (LeFevre, 2014; Reimerink et al., 2013). A high male rAAA mortality rate is significant, as AAA is often asymptomatic and gives no warning before a potentially fatal rupture (Kumar et al., 2017). Generally, once rAAA occurs, the only chance for survival is emergency repair surgery (Guirguis-Blake et al., 2019; Wanhainen et al., 2020).

## Diagnosis, Surveillance, and Treatment

AAA can be reliably diagnosed with imaging modalities including ultrasonography (Benson et al., 2018). Ultrasound has a high specificity (almost 100%) and sensitivity (95%) for visualizing the aorta and detecting AAA (Keisler & Carter, 2015). In addition, ultrasound is safe, inexpensive, and commonly used for diagnosing AAA (LeFevre, 2014; Schaeberle et al., 2015). If an AAA of 3.0 cm or larger is detected, the patient should be formally diagnosed and undergo surveillance every 3 to 12 months (LeFevre, 2014). During the surveillance period, conservative medical treatment and risk factor modification, such as smoking cessation, are standards of care (Isselbacher, 2005). An AAA diameter of 5.5 cm or greater is used as a threshold for surgical repair of AAA to prevent rAAA (Keisler & Carter, 2015; LeFevre, 2014). The leading options for AAA repair are open surgical repair and endovascular aneurysm repair (EVAR) (Benson et al., 2018; Carino et al., 2018). Both surgical methods are used for the elective repair of large AAAs and the emergent repair of rAAAs (Keisler & Carter, 2015).

## Evidence-Based Guidelines

Four major randomized control trials (RCT) have evaluated the effects of one-time screening for AAA with ultrasound in asymptomatic men aged 65 or older (Lindholt et al., 2005; Norman et al., 2004; The Multicentre Aneurysm Screening Study Group, 2002; Wilmink et al., 1999). All four trials exhibited lower AAA mortality, rAAA incidence, emergency repair surgeries, and 30-day postoperative mortality in intervention cohorts screened for AAA compared to control cohorts not screened for AAA (Lindholt et al., 2005; Norman et al., 2004; The Multicentre Aneurysm Screening Study Group, 2002; Wilmink et al., 1999). A meta-analysis of population based RCTs estimated that inviting men 65 years and older to screen was associated with decreased AAA-related mortality and AAA-related ruptures over 12–15 years (Guirguis-Blake et al., 2019). Based on the findings of an RCT that also compared AAA screening in women, many international guidelines support AAA screening for men only (Stather et al., 2013; Wilmink et al., 1999). Preventative health-care task forces from countries including Canada and the United States issued recommendations on screening for male AAA. The Canadian Task Force on Preventative Health Care recommended one-time screening using ultrasound for men aged 65–80 years old for AAA (Singh et al., 2017). The US Preventative Services Task Force recommended one-time AAA screening using ultrasound for men aged 65–75 years old who have ever smoked at least 100 cigarettes (LeFevre, 2014; Owens et al., 2019).

## Men’s Participation in Screening Programs

From this evidence, it is apparent that AAA screening is an essential men’s preventative health measure that is underutilized in North America and requires attention from policymakers and primary care practitioners (PCPs). Despite guidelines, participation in screening among men remains low in the United States. For instance, Olchanski et al. (2014) reported that less than 1% of eligible men were screened for AAA. To date, there are no provincial or federal AAA screening programs for men in Canada (Ali et al., 2016). Men’s participation in international AAA screening programs varies significantly; in the United Kingdom and Sweden, participation is approximately 80% for older men (Benson et al., 2018; Zarrouk et al., 2013). Interestingly, the United States, the United Kingdom, and Sweden all have AAA screening programs for eligible men; yet participation rates are significantly lower in the United States. Although the United States retains a user-pay health-care system, Medicare has been covering the costs of AAA screening for eligible men since 2007 (Medicare, n.d.). However, the logistics employed by screening programs also vary by country. In the United States and Canada, men are referred to AAA screening by their physician; in Sweden, electronic population-based invitations are sent to eligible men recommending their participation, bypassing physicians (Hultgren et al., 2020).

The Canadian and U.S. AAA screening guidelines provide recommendations to PCPs, because preventative medicine is a core practice in primary care (Canadian Medical Association, 2019; LeFevre, 2014; Singh et al., 2017). But, given the discrepancies between participation rates in North America and Europe, a practice gap likely exists in AAA screening for men and by extension PCP compliance with guidelines. The purpose of the current scoping review is to provide a synthesis of the literature to describe the benefits and potential harms of screening older North American-based men (≥65 years old) for AAA. In discussing the findings drawn from the literature, additional PCP recommendations are made.

## Methods

The current study was directed by the following research question: What are the benefits and potential harms of screening older adult men for AAA? Arksey and O’Malley’s (2005) scoping review framework neatly matched the aim of the current study wherein summarizing and disseminating key research findings and identifying research gaps were central to synthesizing understandings about AAA screening in men. Scoping review methods utilize a structured approach for extracting key concepts from the available evidence (Mays et al., 2001). The sequence of steps as described by Arksey and O’Malley (2005) were followed: (1) identify the research question, (2) identify relevant studies, (3) select articles and studies, (4) chart the data, and (5) collate, summarize, and report the results.

### Study Selection

CINAHL, MEDLINE Ovid, Embase, and Web of Science databases were used to search and locate relevant studies to address the aforementioned research question. Within these databases, the following keywords were used: *abdominal aortic aneurysm, aortic rupture, health screen, health status indicator, screen, screening, mass screen, screening test, male*, and *men*. These search terms were used in various combinations and as subject headings. Boolean operators “AND” and “OR” were applied to combine search terms and retrieve relevant results.

**Figure 1.**
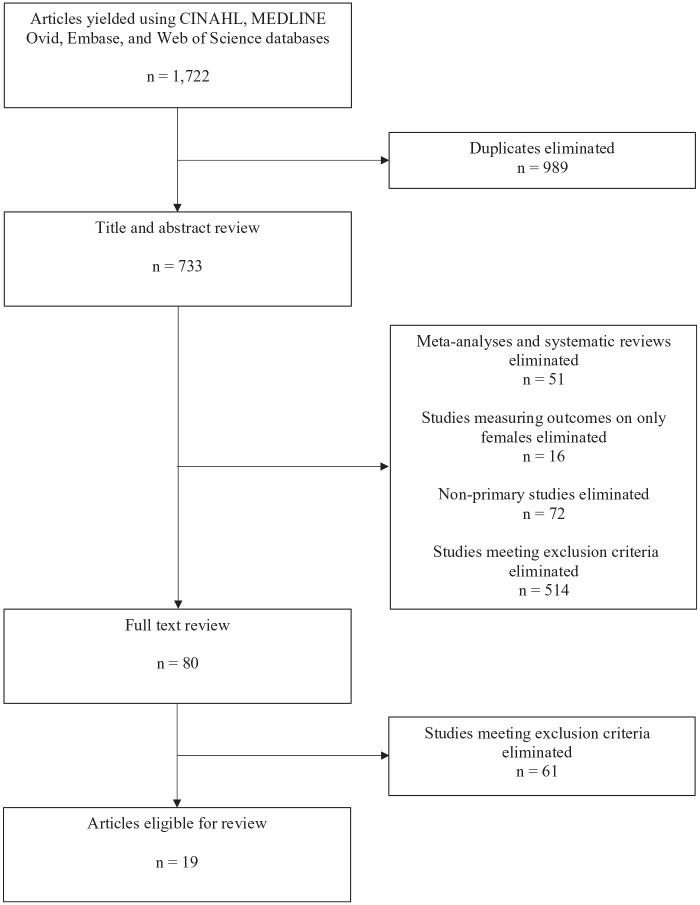
Article Selection Process.

Articles met the inclusion criteria if they were primary empirical studies published in English from 2013 to 2019 inclusive and reported AAA ultrasound screening practices and outcomes in men. Articles were not limited by country of origin. A total of 1722 articles were retrieved in the database searches. In addition to reviews and meta-analyses, studies were also excluded if they did not report screening outcomes in men or disaggregate gender in the study findings, focused on aspects of AAA other than screening (surgical options, etiology, etc.) or technical aspects of sonography, or reported on conditions other than AAA. By eliminating duplicate articles and reading titles and abstracts, followed by full-text reads, 19 articles were ultimately selected for inclusion in the current scoping review (Figure 1).

### Charting the Data

The matrix method, as described by Garrard (2017), was used to organize and review the set of articles (Table 1). The 19 articles were comprehensively read, deconstructed, and their content extracted to build the synthesis matrix and to enable comparison of studies. This allowed key themes to be inductively derived and synthesis of the articles to be produced. These insights are featured in the findings and subsequently discussed in terms of their application to practice.

**Table 1.** Review Articles (*N* = 19).

Author, Year, Location	Aim(s) of Study	Study Design/Sample/Methods	Key Findings
Bath et al., 2018, United Kingdom	To assess the impact of AAA diagnosis on QoL using data from an established AAA screening program.	A prospective cohort study of mental and physical QoL scores for men diagnosed with AAA through participation in AAA screening programs was compared with no-AAA controls (*n* = 5011).	Screening for AAA transiently reduces mental QoL and consistently reduces physical QoL compared with controls. Participants thought about their AAA and the AAA growth progressively less 12 months after the initial screening diagnosis. AAA growth rate had no influence over QoL parameters.
Chan et al., 2019, New Zealand	To describe the proportions of persons dying from AAA who might have benefited from a screening program	Retrospective cross-sectional review of deaths registered in New Zealand from 2010 to 2014, including a subgroup of men. Deaths from AAA were identified from national mortality collection and based on ICD codes and assigned M3 index scores.	Authors dispute the claim that AAA screening is cost-effective as long as prevalence is above 0.35% and argue that relevant costs are not captured in modelling, and cost-effectiveness is over-estimated. Their results support Johansson et al.’s (2018) study: screening programs are likely to have limited impact for a number of reasons, especially given that in this study 77% of deaths had comorbidities and 31% had received an AAA diagnosis prior to the death event.
Chun et al., 2019, United States	To evaluate 10-year outcomes of an AAA screening program in a regional Veteran Affairs health-care system. (Continues work of Chun et al., 2013)	Retrospective chart review of all patients screened for AAA from 2007 to 2016 within a regional Veterans Affairs health-care system identifying men 65 to 75 years of age who smoked a minimum of 100 cigarettes in their lifetime and underwent AAA screening (*n* = 19,626 men) from Jan 1, 2007 to Dec 31, 2016. 66% white ethnicity. 9916 new patients screened from 2012 to 2016.	Overall, 10-year rate of AAA diagnosis was 6.3% or 1232 aneurysms (decline from 7.2% in first 5 yrs). 54 patients with AAA >5.5 cm underwent successful elective repair, a benefit of the AAA screening program. More smaller aneurysms (3.0–4.4 cm) detected and fewer large AAAs >5.5 cm in the last 5 years compared with first 5 years of the program. Increased surveillance exams could burden institutions within the next decade as this AAA cohort of patients (3.0–4.4 cm) grows and the number of large aneurysms (>5.5 cm) decreases. Authors assume cardiovascular risk factor management, smoking cessation, and statins will be treatment approach of most physicians. Average age of screening patients decreased over the 10 year program, suggesting greater awareness, trend for younger men screening, and detection of aneurysms at smaller sizes. Majority of AAA patients (65.7%) screened in 2007 had an average follow-up of 10.2 years. Suspect these patients will outlive their projected 11-year life expectancy, given longer analysis period of 15–20 years. Cannot conclude AAA screening reduces all-cause mortality, but effectiveness of screening, even at 10 years, benefits patients in the long term.
Chun et al., 2013, United States	To evaluate the 5-year outcomes of an AAA screening program in a regional Veterans Affairs health-care system.	A retrospective cohort study using data extracted from a regional Veterans Affairs health-care network identifying all veteran males 65–75 years of age who smoked at least 100 cigarettes during their lifetime and underwent AAA screening (*n* = 9751).	A large AAA screening program at the Veterans Affairs detected more aneurysms, and at smaller diameters than that published in clinical trials. The number of inappropriate AAA screenings has continued to decrease, demonstrating greater awareness and application of the AAA screening guidelines by primary care providers.
Engelberger et al., 2017, Switzerland	To assess the feasibility, acceptance, and costs of an ultrasound scan screening program for AAA in the elderly male population	A prospective cohort study of male patients aged 65–80 years, who attended the outpatient clinics of the Lugano Regional Hospital in 2013, were invited for an ultrasound examination of their abdominal aorta (*n* = 1634).	Screening men aged 65–80 for AAA is feasible, requiring limited financial and organizational effort. Area of residence had a statistically significant impact on attendance.
Ericsson et al., 2017, Sweden	To investigate the psychosocial consequences and SOC in 65-year-old men diagnosed with AAA and participating in a national screening program compared with men with no AAA.	A prospective cohort study using a questionnaire including the Short Form 36 Health Survey, Hospital Anxiety and Depression Scale, SOC, questions concerning stress, and questions related to AAA were answered at baseline and after 6 months after screening (*n* = 170).	Men with AAA reported more problems with physical functioning, pain, stress related to disease, and general health than men with a normal aorta at baseline. No differences were observed between groups in SOC, anxiety, and depression. Having knowledge about the AAA diagnosis may moderately impact physical health and perceived stress.
Glover et al., 2014, United Kingdom	To re-estimate the cost-effectiveness of AAA screening using the most up-to-date available data.	A retrospective cohort study of the existing published model recalibrated to mirror the 10-year follow-up data from MASS. The main cost, AAA growth and rupture rates, and parameters from NAAASP^1^ were re-estimated.^2^	Increased costs and lower AAA prevalence, which increased the cost per QALY. AAA screening is still highly cost-effective.
Hager et al., 2017, Sweden	To evaluate whether screening for AAA among 65-year-old men is cost-effective based on contemporary data on prevalence and attendance rates from an ongoing AAA screening program.	A retrospective cross-sectional study using a decision-analytic model was updated using data collected from an implemented screening program as well as data from contemporary published data and the Swedish register for vascular surgery.^1^	Despite the reduction of AAA prevalence and changes in AAA management over time, screening 65-year-old men for AAA still appears to yield health outcomes at a cost below conventional thresholds of cost-effectiveness.
Johansson et al., 2018, Sweden	To estimate the effect of AAA screening in Sweden on disease-specific mortality, incidence, and surgery.	A retrospective cohort study using data on the incidence of AAA, AAA mortality, and surgery for AAA in men aged 65 years. Men invited to screening between 2006 and 2009 were compared to an age-matched cohort who were not invited for AAA screening (*n* = 131,352).	An insignificant reduction in AAA mortality associated with screening. Screening was associated with increased odds of AAA diagnosis and an increased risk of elective surgery. AAA screening in Sweden did not contribute substantially to the large observed reductions in AAA mortality.
McCaul et al., 2016, Australia	To assess the influence of screening for AAAs in men aged 64–83 years on mortality from AAAs.	A randomized controlled trial of men aged 64–83. The intervention group received invitation for ultrasonography of the abdominal aorta and the control group did not receive an invitation (*n* = 38,480).	There were more elective operations and fewer ruptured AAAs (statistically significant) in the invited group compared to the control group Screening for AAAs had no statistically significant effect on the overall AAA mortality.
Nair et al., 2019, New Zealand	To assess the cost-effectiveness of a UK-style screening program in a New Zealand setting.	Cost comparison of a formal AAA screening program (one-off abdominal ultrasound imaging for about 20,000 men aged 65 years in 2011) with no systematic screening. Markov macrosimulation model was adapted to estimate the health gains (in QALYs), health system costs and cost-effectiveness in New Zealand.	UK-style AAA screening program would be cost-effective in New Zealand. Over the target population of approximately 20, 000 men aged 65 years in 2011, the mean incremental health gain was 117 (95% UI 53 to 212) QALYs. This translates to a mean health gain of 0.006 QALYs per person in the target population. At a cost-effectiveness threshold of NZ $45,000 (£22,100) per QALY (New Zealand’s GDP per capita per QALY), a population-based screening program for AAAs would be cost-effective, even at the upper limit of the uncertainty interval.
Oliver-Williams et al., 2019, UK	To determine the safety of men in the surveillance program after screening and diagnosis of a small or medium AAA by studying the fatal and nonfatal ruptures in this cohort.	Retrospective analyses of men in the national AAA screening program. *N* = 18,652 men screened with an initial AAA of ≥3.0 cm in NAAASP from 2009 to 2017 were followed up.	Cumulative incidence of rupture over 8 years was very low (0.4%). Authors conclude that men enrolled in an intensive surveillance program such as NAAASP are safe, and there is no evidence that the current NAAASP referral threshold of 5.5 cm should be changed. 31 men had a ruptured AAA during surveillance; 29 of these men died. The cumulative incidence of rupture during surveillance reached 0.62% in men with medium aneurysms at baseline and 0.35% for men with small aneurysms at baseline.
Otterhag et al., 2016, Sweden	To evaluate whether screening for AAA has led to a decrease in rAAA incidence.	A retrospective cohort study using data from a Malmö hospital was evaluated for the incidence of rAAA and elective AAA repair 4 years before and after the implementation of a screening program. The Malmö population 4 years before screening implementation (*n* = 285,514) and 4 years after implementation (*n* = 307,207) were evaluated.	In older adult men, the incidence of rAAA decreased (statistically insignificant) and elective AAA repair increased 4 years after the start of a screening program.
Peek et al., 2016, New Zealand	To compare the hospital costs of AAA repair in emergency and elective cases over a 3-year period in a single center in New Zealand.	A retrospective cohort study of consecutive patients undergoing elective and emergency AAA repair (*n* = 169).	rAAA repairs were more expensive than elective AAA repairs, despite no difference in length of hospital stay.
Pettersson et al., 2017, Sweden	To describe how men diagnosed with AAA discovered by screening experience the process and diagnosis from invitation to 1 year after screening.	A qualitative explorative study of eleven 65-year-old men who participated in three focus group interviews (*n* = 11).	Men experienced a lack of health-care support. Men were insecure about how they could adjust their lifestyle to improve their health condition. It is important to communicate knowledge about the AAA to promote men’s feelings of security and give space to discuss the size of the aneurysm and lifestyle changes.
Svensjö et al., 2013, Sweden	To determine the fate of a 65-year-old male population 5 years following an invitation to an aortic ultrasound examination.	A prospective cohort study of men invited to ultrasound examination at age 65 (*n* = 3268) and re-invited at age 70 (*n* = 2811). Mortality, AAA repair, and risk factors were recorded.	AAA screening in a contemporary setting was safe at 5 years. Men with a screening detected AAA had a high repair rate and high non-AAA related mortality.
Svensjö et al., 2014, Sweden	To assess the clinical and health economic effectiveness of one-time screening of 65-year-old men.	A prospective cohort study comparing one-time ultrasound screening of 65-year-old men (invited) versus no screening (control). Data were analyzed in a Markov model. ^1^	Screening men for AAA remains cost-effective. Absolute and relative risk reductions were noted since the recent changes in the management and epidemiology of AAA.
Wanhainen et al., 2016, Sweden	To determine the outcome of an AAA screening program targeting men aged 65 years.	A prospective cohort study using data from all Swedish counties to measure the number of invited and examined men, screening- detected AAAs, AAAs operated on, and surgical outcomes (*n* = 302,957).	The introduction of screening was associated with a significant reduction in AAA-specific mortality. Screening 65-year-old men for AAA is an effective preventive health measure and is highly cost-effective in a contemporary setting.
Zucker et al., 2017, United States	To assess changes in AAA ultrasound screening associated with the release of revised 2-14 USPSTF recommendations.	A retrospective cohort study of AAA screening ultrasound examinations performed in the Massachusetts General Hospital radiology department in the 15 months before and after the new guidelines were reviewed (*n* = 831).	The updated USPSTF^3^ guidelines have been associated with increased AAA screening appropriateness and aneurysm detection in our practice, with smaller aneurysm size at diagnosis.

*Note.* AAA = abdominal aortic aneurysm; QoL = quality of life; QALY: quality-adjusted life-year.

1 National Abdominal Aortic Aneurysm Screening Program.

2 Various sample sizes utilized for different outcome measurements and analyses.

3 U.S. Preventive Services Task Force.

## Findings

Of the 19 articles included in the current scoping review, 18 employed quantitative analysis methods and one used a qualitative approach (Pettersson et al., 2017) (Please see Table 1). Among the 18 quantitative studies, six were prospective cohort studies, six were retrospective cohort studies, three were retrospective cross-sectional studies, one was a retrospective mortality study, one was a comparative cost analysis, and one was an RCT. The qualitative study employed an exploratory design methodology, using focus group interviews. Eight studies were conducted in Sweden, three in the United States, three in the United Kingdom, one in Switzerland, three in New Zealand, and one in Australia. Overall, findings from 12 articles supported AAA screening, five studies opposed AAA screening, and two articles were somewhat balanced in their benefits and harms findings. The findings drawn from the analyses are organized under two descriptive labels: (1) benefits of AAA screening for men, and (2) potential harms of AAA screening for men. Thematic findings developed within the benefits included: (a) reducing mortality, and (b) cost-effectiveness. The two potential harm themes were: (a) lack of mortality and morbidity benefits, and (b) inconsistent application of AAA screening recommendations.

### Benefits of Male AAA Screening

#### Reducing Mortality

The screening detected point prevalence of AAAs in men varied from 1.5% to 7.1% (Chun et al., 2013; Engelberger et al., 2017; McCaul et al., 2016; Svensjö et al., 2013; Wanhainen et al., 2016). Among these screen-detected AAAs, 0.4%–7% were large aneurysms (greater than 5.5 cm) (Chun et al., 2013; McCaul et al., 2016; Wanhainen et al., 2016). In one study, the new diagnosis of AAAs in 65-year-old men was attributed to screening in 98% of cases (Svensjö et al., 2013). The authors attributed the increased detection and smaller AAA diameter size at detection to updated U.S. guidelines (Zucker et al., 2017). Confirming this trend, the 10-year outcome evaluation of the American veterans’ AAA screening program reported fewer large AAAs had been detected in the last 5 years of the study, and overall, more but smaller (3.0–4.4 cm) aneurysms were being detected. In addition, the 10-year rate of AAA diagnosis had declined from 7.2% to 6.3% in the first 5 years with patients expected to outlive their predicted 11-year life expectancy (Chun et al., 2019).

In a prospective study, zero men with screen-detected AAAs experienced rAAA, and the only documented rAAA case occurred in a cohort of men that did not attend AAA screening (Svensjö et al., 2013). Also reported was a statistically significant lower incidence of rAAA in a screened group (*n* = 72; 0.37%) versus a non-screened group (*n* = 99; 0.51%) (McCaul et al., 2016). Four years after the initial implementation of a Swedish national screening program, the incidence of rAAA in men decreased from 10.5 to 6.2 per 100,000 person-years (Otterhag et al., 2016). A UK retrospective study of men in their national AAA screening program reported that the cumulative incidence of rupture over 8 years (2009–2017) was very low (0.04%) (Oliver-Williams et al., 2019). Thirty-one men experienced a ruptured AAA and 29 died; however, the authors concluded men enrolled in intensive surveillance were safe, and that the 5.5-cm threshold for referral should not be changed (Oliver-Williams et al., 2019).

Screening men for AAA can contribute to decreased mortality from fatal rupture (Oliver-Williams et al., 2019). AAA mortality was approximately 25% lower in men who were screened compared to men who were not screened for AAA (Johansson et al., 2018; Wanhainen et al., 2016). A study of AAA screening among men 65 years and older reported mortality rates 2.4 times higher in a group that did not attend screening versus a group that attended (Svensjö et al., 2013). A significant AAA mortality reduction was noted in Swedish counties that had established screening programs for greater than 6 years versus regions that had programs for less than 4 years—a mean AAA-mortality reduction of 4% for every year screened (Wanhainen et al., 2016). AAA mortality for men ≥65 years old was 38–74 per 100,000 person-years in a group not screened for AAA, and 35–45 per 100,000 person-years in a group invited to AAA screening (McCaul et al., 2016; Wanhainen et al., 2016). In Western Australia, AAA mortality in men who attended AAA screening was reduced by half, attributed to early detection and intervention (McCaul et al., 2016).

After 4 years of a national Swedish screening program, absolute risk of AAA mortality decreased from 1.3 % to 0.3% deaths with two in-hospital rAAA, which translates to a relative risk reduction of 75% (Otterhag et al., 2016). After 13 years of inviting 65-year-old men to AAA screening, the relative risk reduction of AAA mortality was 40%–42%, and the absolute risk reduction was approximately 15.1 per 10,000 men invited (Svensjö et al., 2014; Wanhainen et al., 2016). Wanhainen et al. (2016) estimated that AAA screening prevented 90 premature AAA deaths annually. Numerous studies in the current scoping review support the assertion that early detection of AAA from screening older men is associated with a decreased incidence of rAAA, thus reducing mortality (McCaul et al., 2016; Oliver-Williams et al., 2019; Otterhag et al., 2016; Svensjö et al., 2013, 2014; Wanhainen et al., 2016).

#### Cost-Effectiveness

Detecting AAA by screening older men can allow for efficient repair using elective surgery, which reduces the financial burden of emergency AAA repair surgeries. Of 9751 men aged 65–75 who were screened for AAA in the United States, 67.4% underwent elective repair surgery for large AAA (Chun et al., 2013). In Sweden, the incidence of elective AAA repair surgery in men aged 60–69 before and after the implementation of a national screening program was 9.7 and 44.2 per 100,000 person-years, respectively (Otterhag et al., 2016). This study reported that the ratio of emergency versus elective AAA repair surgery for men was 65.5% (58:38) before and 22.7% (75:17) after the implementation of a screening program (Otterhag et al., 2016). McCaul et al. (2016) identified significantly more cases of elective AAA repair surgeries in a cohort of men aged 64–83 invited for AAA screening (*n* = 536) compared to men who were not invited (*n* = 414). Several studies confirmed that 18%–50% of AAAs electively repaired were detected from screening (Otterhag et al., 2016; Svensjö et al., 2013; Wanhainen et al., 2016). Furthermore, the median length of stay in hospital or a rehabilitation facility was 7–8 days after elective repair and 10–12 days after emergency repair, resulting in higher costs for emergency repairs within inpatient settings (Peek et al., 2016).

Wanhainen et al. (2016) reported a reduction of emergency rAAA repairs after the implementation of a national Swedish AAA screening program. Long-term predictions were an annual caseload of 360 elective AAA repairs (109% more than with no screening) and 36 rAAA repairs (59% less than with no screening) in men aged 65 years (Wanhainen et al., 2016). In an RCT, 30-day mortality after AAA-repair surgery was 2.4% in men who attended AAA screening, and 4.1% in men who were not offered screening (McCaul et al., 2016).

Screening older adult men for AAA was described as cost-effective in contemporary epidemiologic and economic climates (Glover et al., 2014; Hager et al., 2017; Svensjö et al., 2014; Wanhainen et al., 2016). Each U.S.-based AAA ultrasound screening examination cost $58 in 2008 and $38 in 2012; therefore, the long-term implementation of an AAA screening program was predicted to become more cost-effective over time (Chun et al., 2013). In addition, screening programs that decreased the incidence of rAAA would consequently reduce inpatient hospital costs from emergency AAA repairs (Peek et al., 2016).

Quality-adjusted life-year (QALY) is a measurement of a person’s years of life adjusted to reflect their quality of life (QOL): one QALY equals one year in perfect health (National Institute for Health and Care Excellence [NICE], n. d.). A standard threshold for the effective usage of health-care resources is US$26,277–US$39,416 per QALY (Glover et al., 2014; Hager et al., 2017; National Institute for Health and Care Excellence, 2013). The cost of extending one man’s life with an invitation to AAA screening varied from US$6997 to US$16,270 per QALY (Hager et al., 2017; Svensjö et al., 2014; Wanhainen et al., 2016). Within the studies included in the current review, all costs per QALY were well below the NICE threshold of US$26,277. The cost per life-year (LY) gained from screening older adult men for AAA was US$5346–US$12,787 (Glover et al., 2014; Hager et al., 2017; Svensjö et al., 2014). The 4.8 LYs gained from each rAAA prevented by inviting eligible men to AAA screening was clinically significant in Svensjö et al.’s (2014) study, as life expectancy is increasing, and AAA repair outcomes are improving.

The one-time screening of 65-year-old men for AAA was assessed as cost-effective in the current health-care climate, despite the decreased prevalence of AAA and economic inflation, as it was counterbalanced with better surgical repair outcomes and increased life expectancy (Glover et al., 2014; Hager et al., 2017; Svensjö et al., 2014; Wanhainen et al., 2016). If AAA prevalence were as low as 0.35%–0.5%, a screening program would still be cost-effective (Glover et al., 2014; Svensjö et al., 2014). Nair et al.’s (2019) cost-effectiveness analyses to estimate the QALYs gains and health system costs concluded that a UK-style population screening program would be cost-effective in New Zealand. These findings support the claim that screening older men for AAA is cost-effective and increases the proportion of elective AAA repair surgeries compared to emergency surgeries, which is associated with reduced postoperative mortality (Glover et al., 2014; Hager et al., 2017; McCaul et al., 2016; Nair et al., 2019; Otterhag et al., 2016; Peek et al., 2016; Svensjö et al., 2014; Wanhainen et al., 2016).

### Potential Harms of Male AAA Screening

#### Lack of Mortality and Morbidity Benefits

In contrast to the aforementioned finding of reduced male AAA mortality through screening, some studies in the current review reported no AAA mortality or morbidity screening-related benefits (Chan et al., 2019; Johansson et al., 2018; McCaul et al., 2016; Otterhag et al., 2016). These authors argued that with decreasing prevalence rates of AAA and AAA mortality and comorbidities in older adult men, the benefits of screening can become less pronounced. Johansson et al. (2018) pointed out that AAA mortality rates began to decrease a decade before the implementation of a national screening program and continued to decrease at the same rate after the start of a screening program. AAA mortality decreased by over 70% in men aged 65–74 years old, without a noticeable difference between invited and noninvited cohorts (Johansson et al., 2018). One RCT reported screening men for AAA resulted in an insignificant reduction of AAA mortality during a 13-year-follow-up period after the implementation of a screening program (McCaul et al., 2016). Additionally, men aged 65–74 who belonged to the screening group in this study had an 8% lower AAA mortality than those in the no-screening group, which is considerably lower than the 42% AAA mortality reduction in one of the main trials that informed the Canadian and U.S. guidelines (McCaul et al., 2016; The Multicentre Aneurysm Screening Study Group, 2002).

A Swedish study reported a contemporary decrease of rAAA prevalence in men before the initiation of AAA screening programs, and no significant reduction of rAAA mortality in screened men 4 years after the implementation of an AAA screening program (Otterhag et al., 2016). Another study identified increased 30-day mortality after rAAA in a cohort of men invited for screening (61.5%) compared to a cohort not invited for screening (43.2%) (McCaul et al., 2016). McCaul et al. (2016) concluded that screening men aged 65–74 years old for AAA was ineffective because AAA mortality was not significantly reduced, due to lower than expected AAA mortality rates in a cohort of non-screened men. Related to this issue, Chun et al. (2019) predicted that because more men were being detected with smaller AAAs at younger ages, increased annual surveillance would likely burden health institutions in the United States. Furthermore, Johansson et al. (2018) doubted the benefits of an AAA screening program, as decreasing rates of rAAA and AAA mortality were seen in the older adult male population, regardless of screening. In a 2010–2014 New Zealand death register study, the authors disputed claims for screening programs being cost-effective based on their findings that 77% of the AAA deaths had life limiting comorbidities (i.e., cancer and/or cardiovascular disease) and 31% had been diagnosed with AAA diagnosis prior to their death (Chan et al., 2019). Therefore, the proposed benefits of screening older adult men for AAA are debatable in the setting of declining AAA mortality and rAAA prevalence unrelated to screening (Johansson et al., 2018; McCaul et al., 2016; Otterhag et al., 2016).

Three studies exhibited a negative impact on men’s QoL after undergoing AAA screening. QoL outcomes, measured as physical component summary (PCS) scores, showed significantly lower scores at 37 months post-screening in a cohort of men diagnosed with screening-detected AAA versus a cohort without AAA, indicating that these men had a consistently lower physical QoL (Bath et al., 2018). Compared to men without a screening-detected AAA, men with AAA reported significantly higher levels of disease-related stress six months after screening (Ericsson et al., 2017). Ericsson et al. (2017) concluded that men who underwent AAA screening, regardless of receiving an AAA diagnosis, reported a significant decrease of physical and emotional role functioning, physical functioning, mental functioning, social functioning, and low PCS scores 6 months after screening (Ericsson et al., 2017).

In a qualitative focus group study, men with screening-detected AAA expressed varying levels of anxiety (Pettersson et al., 2017). Many men were unaware that an AAA detected during screening may require life-long surveillance (Pettersson et al., 2017). However, most men did not trust the reliability of their AAA measurement. For example, one man was initially diagnosed with an AAA at screening, but his aortic diameter was within normal limits at follow-up (Pettersson et al., 2017). Some men did not have a health-care professional to consult after being diagnosed with AAA and felt answers from physicians were unclear (Pettersson et al., 2017).

Men worried about their AAA growing, which caused anxiety and negative impacts on their lives, including concern that they could not control the disease course (Ericsson et al., 2017; Pettersson et al., 2017). Some men had uncertainties about how their lifestyle would affect their AAA and limited their physical activity out of fear of rupturing their AAA (Pettersson et al., 2017). Two men expressed living with AAA was a death threat, stating, “over 50 . . . then it’s critical that it can burst anytime,” and “41-55, well I have 14 mm left until death” (Pettersson et al., 2017, pp.73–74). In summary, findings from studies in the current review assessing the consequences of AAA screening on men’s QoL suggest that screening was associated with a negative psychosocial impact on men, and these QoL implications should be considered when implementing screening programs, as ostensibly healthy patients can be diagnosed with a lifelong condition (Ericsson et al., 2017; Pettersson et al., 2017).

#### Inconsistent Application of AAA Screening Recommendations

AAA screening eligibility is recommended for men ≥65 years old (LeFevre, 2014; Singh et al., 2017); however, inconsistent application of AAA screening recommendations by PCPs, and the associated possibility of false positives and over- or under-treating was a caveat to screening older men for AAA (Chun et al., 2013; Zucker et al., 2017). In the United States, 25%–28.2% of individuals who were screened for AAA did not meet the inclusion criteria outlined by guidelines (Chun et al., 2013; Zucker et al., 2017). Of the appropriately screened patients, 1.3% without screening-detected AAAs had multiple examinations, 100% of those with inconclusive results had no follow-up scans to rule out AAA definitively, and 34.1% with screening-detected AAAs had no follow-up surveillance examinations (Chun et al., 2013). Presumed explanations for poor surveillance adherence were lack of PCP awareness of surveillance protocols, appointment cancellations, and rewarding PCPs for screening without penalizing them for inappropriately screening men that do not meet the inclusion criteria (Chun et al., 2013).

Johansson et al. (2018) also reported rates of AAA false positives among men and overtreatment as a result of screening; however, this study had a smaller sample size and follow-up period compared to Wanhainen et al. (2016), another Swedish study included in the current review. According to this study, there was a false positive rate of 49 per 10,000 (0.49%) for AAA from screening (Johansson et al., 2018). The odds of having elective AAA repair surgery were higher in a cohort of men invited to screening, resulting in 19 per 10,000 (0.19%) men potentially overtreated because the increase in repairs did not correlate with decreased rAAA cases (Johansson et al., 2018). As demonstrated in these findings, screening inappropriate patients can contribute to the potentially harmful aspects of screening older adult men for AAA and reduce cost-effectiveness of a screening program (Chun et al., 2013; Johansson et al., 2018; Zucker et al., 2017).

## Discussion

The current scoping review provides a synthesis of the literature to describe benefits and potential harms of screening older (≥65 years old) North American men for AAA. The benefits of screening older adult men for AAA were (a) decreased AAA-related mortality, and (b) proven cost-effectiveness of a screening program. The potential harms associated with screening older men for AAA were (a) lack of mortality and morbidity benefits, and (b) inconsistent application of AAA screening recommendations. Although the potential harms of AAA screening were highlighted by five studies, the benefits of screening dominated the findings of the current scoping review. Based on the findings drawn from the current scoping review, AAA screening guidelines are discussed, along with suggested changes to reflect current AAA epidemiology, improved translation of evidence to practice and policy, and increased PCP compliance to screening older adult men for AAA.

Screening older adult men for AAA has increased rates of AAA detection, decreased rAAA prevalence, decreased AAA mortality, increased elective AAA repair, and decreased emergency AAA repair (McCaul et al., 2016; Otterhag et al., 2016; Peek et al., 2016; Svensjö et al., 2013; Wanhainen et al., 2016). There has been an overall decline in AAA prevalence, rAAA prevalence, and AAA mortality, regardless of AAA screening (Johansson et al., 2018; McCaul et al., 2016; Otterhag et al., 2016). AAA screening guidelines from Canadian and U.S. preventative health-care task forces are informed by four major trials published between 1999 and 2005 that are likely outdated (Johansson et al., 2018; Lindholt et al., 2005; Norman et al., 2004; The Multicentre Aneurysm Screening Study Group, 2002; Wilmink et al., 1999). To empirically inform updated guidelines, new trials studying the outcomes of screening older men for AAA within the current epidemiological climate should be completed. Since findings in the current review demonstrate a decline in AAA prevalence in men, guidelines should reflect the need for PCPs to evaluate older men individually for AAA screening appropriateness, by taking personal risk factors and estimated life expectancy into account (Olchanski et al., 2014). For instance, men with a higher number of comorbid conditions, such as acquired immunodeficiency syndrome (AIDS), dementia, liver disease, renal failure, heart failure, and chronic obstructive pulmonary disease (COPD), have shorter life spans (Cho et al., 2013). This may translate into the need for a customized AAA screening age range for older adult men (Kapila et al., 2018). To reflect the current epidemiology of AAA in older adult men, the 2019 U.S. Preventive Task Force updated their recommendations by incorporating new evidence into the statement (Owens et al., 2019). However, recommendations are consistent with the previous guidelines suggesting that clinicians selectively offer screening to men 65–75 years who have ever smoked, rather than routinely screening all men in this age group. This recommendation guides U.S. clinicians to individually consider each male patient’s medical history, values, and risk factors to determine if and when AAA screening is appropriate.

Despite evidence of the benefits and recommendations supporting AAA screening in older men, screening programs remain sparse in North America (Kapila et al., 2018). In addition, screening programs have been questioned based on concerns about false positives and the associated psychological harms of diagnosing healthy older men (Chan et al., 2019; Johansson et al., 2015, 2016). A systematic review, however, concluded there is no evidence that surveilling older men for AAAs negatively impacts their mental health or quality of life (Lyttkens et al., 2020).

To fully realize AAA screening benefits, improved translation of evidence to policy is required. For instance, although improved survival and decreased mortality as a result of screening is indisputable, the tendency in the United States and Australia to repair AAAs smaller than the recommended threshold may threaten benefits and cost-effectiveness (Lederle, 2016). Implementing a widespread AAA screening policy that is patient centered, focused on mortality benefits, and feasible for PCP workflow should be considered by policymakers as it has been proven to be highly cost effective in several European countries (Olchanski et al., 2014; Pettersson et al., 2017; Spronk et al., 2011). As a result of widely accepted AAA screening policies and programs, practicing routine AAA screening for older men may become normalized and the benefits of screening, as shown in the current review, might come to fruition.

From a PCP perspective, low screening rates were due to lack of familiarity with guidelines and prioritizing other screening examinations over AAA (Eaton et al., 2012). To increase PCP awareness of guidelines, preventative health-care task forces and vascular societies must consider strategies to improve guideline dissemination, with emphasis on screening men that meet the inclusion criteria determined by guidelines. Financially incentivizing PCPs may improve compliance; however, this may reduce the overall cost-effectiveness of a screening program (Wanhainen et al., 2016). Another method to aid in increasing AAA screening rates and attendance is best practice alerts, which alert the PCP to screen an eligible patient for AAA when their electronic medical record is opened (Hye et al., 2014). Screening for AAA could also be modelled after breast and colorectal cancer screening programs in Canada and guided by the population-based invitation system employed in Sweden, rather than leaving referrals to individual physicians. Additionally, media campaigns that target men over 65 years and encourage self-referral for AAA screening may achieve a higher AAA detection rate (Meecham et al., 2016). To further reduce costs, strategies should be implemented that aim to decrease the rate of screening patients that do not meet the criteria (Chun et al., 2013; Owens et al., 2019).

Limitations of the current scoping review include the geographical diversity of the included studies. AAA screening programs, guidelines, epidemiology, and costs vary from country to country. In addition to diverse cultural values surrounding men’s health in these jurisdictions, there are considerable system differences for AAA screening in countries such as Sweden and Canada (universal public health system) versus the United States (private user-pay system). Further, studies in this scoping review focused on medical outcomes; however, there are social determinants of health including ethnicity, education, and income that influence screening program access and outcomes that are not addressed herewith. It is also important to note the limits of scoping reviews wherein they are not intended to formally evaluate study designs and methodologies nor assign empirical weights to specific study findings. Due to these discrepancies, the findings of the current review may not be directly comparable across the included studies.

## Conclusion

PCPs must consider the benefits and potential harms when deciding to screen older men for AAA. To aid in this decision-making process, guidelines for PCPs can support consistent AAA screening, diagnostic, and treatment practices. Although the current review found that the benefits of screening for AAA outweigh the potential harms, PCPs must consider each older male patient for AAA screening on an individual basis, as the potential harms cannot be disregarded. Additionally, screening programs should be studied in the context of each region’s AAA/rAAA epidemiology, including cost analyses, before implementation, to determine feasibility. Ultimately, screening men for AAA can allow for early detection, surveillance, and intervention of a potentially fatal condition (Zucker et al., 2017). Screening older adult men for AAA can be a practical preventative approach to men’s health.
